# Sulphate of Nickel—a Sedative

**Published:** 1869-05

**Authors:** 


### Selections.

Sulphate of Nickel—A Sedative.—Dr. J. Dabney Palmer writes, (*Amer. Jour. of Pharmacy*,) Sulphate of Nickel has been "employed in a variety of painful affections and also with the view of simply producing sleep, where Dover's powder and other preparations of opium were contra-indicated, and in every instance produced the desired effect. This experience, although limited, inclines me to believe that the sulphate may be regarded as a valuable sedative, and, unlike many others of that class, it is not followed by disagreeable sensations, nor derangements of the alimentary canal." It is given in half-grain or grain doses three times a day.

